# P-Glycoprotein as a Therapeutic Target in Hematological Malignancies: A Challenge to Overcome

**DOI:** 10.3390/ijms26104701

**Published:** 2025-05-14

**Authors:** Pablo Álvarez-Carrasco, Fernanda Morales-Villamil, Carmen Maldonado-Bernal

**Affiliations:** 1 Laboratorio de Investigación en Inmunología y Proteómica, Hospital Infantil de México Federico Gómez, Mexico City 06720, Mexico; 2 Facultad de Medicina, Universidad Nacional Autónoma de México, Mexico City 04510, Mexico; 3 Facultad de Medicina, Benemérita Universidad de Puebla, Puebla 72000, Mexico

**Keywords:** hematological malignancies, P-glycoprotein, multidrug resistance, therapeutic target

## Abstract

P-glycoprotein (P-gp), a transmembrane efflux pump encoded by the *ABCB1/MDR1* gene, is a major contributor to multidrug resistance in hematological malignancies. These malignancies, arising from hematopoietic precursors at various differentiation stages, can manifest in the bone marrow, circulate in the bloodstream, or infiltrate tissues. P-gp overexpression in malignant cells reduces the efficacy of chemotherapeutic agents by actively expelling them, decreasing intracellular drug concentrations, and promoting multidrug resistance, a significant obstacle to successful treatment. This review examines recent advances in combating P-gp-mediated resistance, including the development of novel P-gp inhibitors, innovative drug delivery systems (e.g., nanoparticle-based delivery), and strategies to modulate P-gp expression or activity. These modulation strategies encompass targeting relevant signaling pathways (e.g., NF-κB, PI3K/Akt) and exploring drug repurposing. While progress has been made, overcoming P-gp-mediated resistance remains crucial for improving patient outcomes. Future research directions should prioritize the development of potent, selective, and safe P-gp inhibitors with minimal off-target effects, alongside exploring synergistic combination therapies with existing chemotherapeutics or novel agents to effectively circumvent multidrug resistance in hematological malignancies.

## 1. Introduction

P-glycoprotein (P-gp), also known as multidrug resistance protein 1 (MDR1), is one of the most studied multidrug resistance transporters and plays a critical role in mediating drug resistance in cancer cells. Encoded by the ABCB1 gene, P-gp functions as a transmembrane efflux pump, actively transporting a wide range of substrates, including chemotherapeutic agents, out of cells. It plays a pivotal role in drug disposition and resistance. This activity significantly influences drug disposition and reduces the efficacy of anticancer therapies [1]. By extruding chemotherapeutic drugs from cancer cells, P-gp contributes to the development of resistance to chemotherapy. P-gp is a member of the ATP-binding cassette (ABC) transporter family, which comprise 48 energy-dependent membrane transport proteins [2].

While the role of P-gp in mediating drug resistance within oncological contexts is well-established, its expression across a diverse array of tissues underscores its broader physiological and pathological significance, serving as an essential protective barrier in key sites like the intestine, kidney, and blood–brain barrier. P-gp controls the disposition of numerous endogenous and exogenous compounds. Recent investigations have highlighted the implication of P-gp in the pathogenesis and therapeutic response of various conditions beyond hematological malignancies, illustrating its influence on drug disposition and treatment efficacy across diverse cellular environments, from lymphocytes to fibroblasts [3,4]. However, despite these observations elucidating the multifaceted impact of P-gp across various pathological states, its involvement in hematological malignancies remains a subject of critical importance. Immune cells within hematological malignancies, such as acute myeloid leukemia (AML), acute lymphoblast leukemia (ALL), diffuse large B-cell lymphoma, multiple myeloma, and follicular lymphoma, demonstrate increased P-gp expression, contributing to chemotherapy resistance. [5,6]. Therefore, while the broader physiological and pathological roles of P-gp are acknowledged, the imperative to further elucidate the mechanisms by which P-gp contributes to drug resistance in these cancers is paramount.

Given the critical role of P-gp in mediating drug resistance within hematological malignancies, as previously discussed, the development of effective P-gp inhibitors has been a significant focus. Numerous first-, second-, third-, and fourth-generation P-gp inhibitors have been developed and tested in various clinical trials [7]. However, despite advancements in inhibitor design, including improved specificity and reduced toxicity in later generations, none have yet achieved successful clinical approval, primarily due to their lack of efficacy in clinical trials and potential for adverse pharmacokinetic interactions when used in combination with other drugs [8,9,10,11]. It is also important to consider the potential impact of P-gp inhibition when evaluating first-line therapies not solely reliant on conventional chemotherapy, as some targeted agents may be P-gp substrates or their efficacy may be modulated by P-gp function. Consequently, this review underscores the urgent need for intensified research into novel strategies for P-gp inhibition, aiming to optimize chemotherapy treatments and other therapeutic modalities specifically within the context of malignant hematological neoplasms. The challenge posed by multidrug resistance, largely mediated by P-gp overexpression, remains a substantial obstacle in the management of these cancers, severely limiting the efficacy of chemotherapeutic agents and negatively impacting patient survival.

## 2. P-Glycoprotein Generalities

As a 170 kD transmembrane protein of the ATP-binding cassette (ABC) superfamily, P-gp actively transports diverse substrates across membranes using ATP. This energy-dependent efflux, extending beyond chemotherapeutic agents, impacts physiological and pathological processes. By reducing intracellular drug concentrations, P-gp directly affects cancer therapeutic efficacy, contributing to multidrug resistance, notably in hematological malignancies [6,12,13].

**Figure 1:**
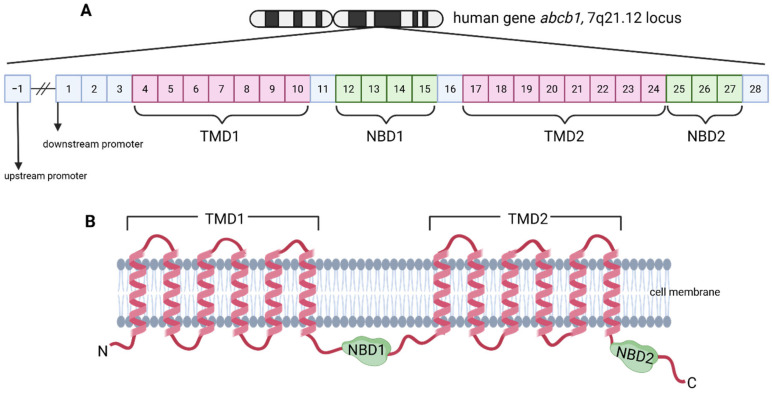
Structure of the human ABCB1 gene and its protein product, P-glycoprotein. (**A**) Schematic representation of the human ABCB1 gene. The gene consists of 28 exons and two promoters: a downstream promoter located within exon 1 and an upstream promoter located proximal to exon −1. Exons 4–10 and 17–24, colored in pink, encode the transmembrane domains TMD1 and TMD2, respectively. Exons 12–15 and 25–27, colored in green, encode the nucleotide-binding domains, NBD1 and NBD2, respectively [15,16]. (**B**) Schematic representation of the P-glycoprotein (P-gp) protein. P-gp is a transmembrane protein with twelve transmembrane helices (six in each transmembrane domain, TMD1 and TMD2) colored in pink. The two nucleotide-binding domains (NBD1 and NBD2), colored in green, are located intracellularly. NBD1 is positioned between TMD1 and TMD2, while NBD2 is located after TMD2. Created in BioRender by Alvarez-Carrasco, P. https://BioRender.com/q92n992 (accessed on 21 December 2024).

The structure of P-gp (Figure 1) reveals a typical ABC transporter fold with two transmembrane domains (TMD) containing twelve transmembrane helices divided into two symmetrical halves by a flexible loop containing two cytoplasmic nucleotide-binding domains (NBDs). This allows for P-gp to undergo dynamic conformational changes to bind and efflux a wide range of hydrophobic and amphipathic drug substrates [6,12].

The flexibility of the linker loop is crucial for the synergy between the two ATP binding sites required for substrate translocation. Each NBD comprises three motifs: Walker A, Walker B, and Signature C. The ATP binding site is made up of the Walker A and Walker B motifs from one NBD and the Signature C motif from the other NBD, with conserved residues aiding in ATP-Mg^2+^ ion binding and subsequent hydrolysis [12,14]. Activation of the P-gp pump necessitates the binding of Mg^2+^ ions to the ATP sites, facilitating ATP hydrolysis for energy transduction to the TMDs [14].

**Figure 2:**
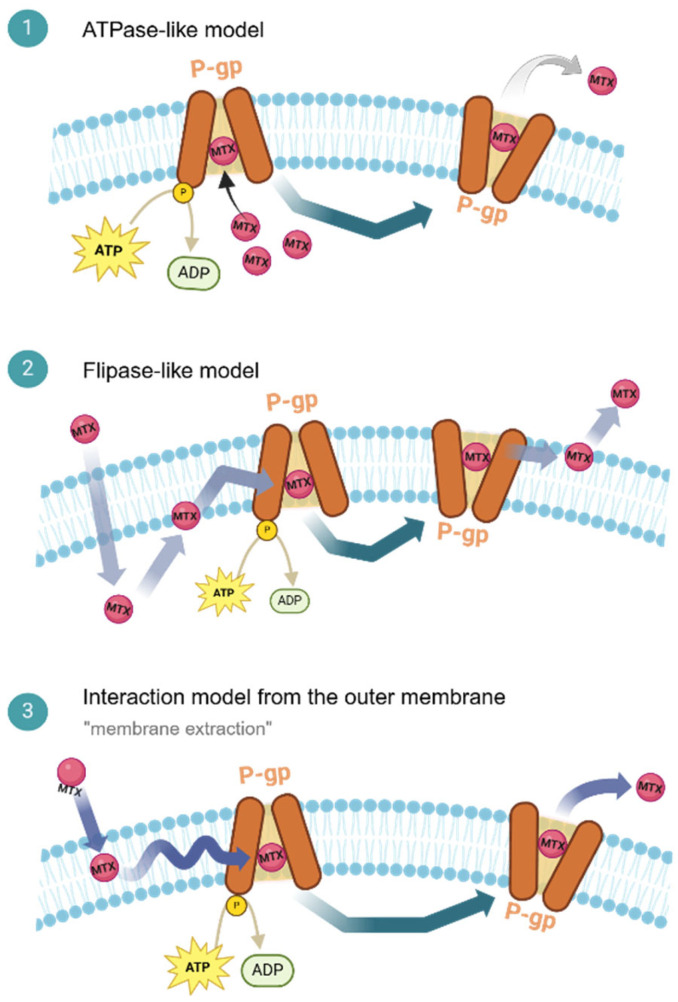
Proposed mechanisms of P-gp-mediated drug efflux. Three primary models have been proposed to explain P-gp-mediated drug efflux: (**1**) direct extraction, where P-gp directly extracts substrates from the cytoplasm; (**2**) flippase activity, where P-gp transports substrates from the inner to the outer leaflet of the plasma membrane; and (**3**) membrane extraction, where P-gp directly removes substrates from the outer leaflet of the membrane. Created in BioRender by Alvarez-Carrasco, P. https://BioRender.com/m52w661 accessed on 14 January 2025.

P-gp recognizes and binds to its substrates through weak electrostatic interactions, such as hydrogen bonding, π-π stacking, and π-cation interactions (Figure 2) [17]. The binding affinity of drugs to P-gp is influenced by their molecular size and the number and type of hydrogen bond acceptor groups they possess. Also, this protein can simultaneously bind to multiple drug molecules, which can lead to the inhibition or induction of its activity, depending on the specific compounds involved [13].

P-gp possesses two primary binding sites with distinct functions:

- The Main Binding Cavity (MBC), situated at the apex of P-gp, is a critical region for substrate binding prior to their efflux from the cell. This cavity plays a central role in the interaction with a diverse array of compounds, encompassing both substrates and non-substrates of P-gp. Specifically, the MBC exhibits a preferential affinity for substrates, facilitating their subsequent transportation to the extracellular environment. Compounds classified as strong substrates, defined by an efflux ratio (ER) exceeding 2, demonstrate a particularly high affinity for this binding site [18].
- Distinct from the MBC, located at the apex of P-gp, Other Binding Sites (OBS) are situated within the middle region of the protein. These sites are primarily for interacting with non-substrates, compounds that are not effluxed by P-gp, thus facilitating their entry into the cell. Non-substrates, characterized by an ER of less than 1, preferentially bind to the OBS. Compounds exhibiting intermediate ER values (between 1 and 2) demonstrate a more balanced binding affinity, distributing relatively equally between the MBC and the OBS [18].

In this study, the ER serves as a key metric for quantifying the interaction of compounds with P-gp and their propensity for active efflux. Compounds are categorized based on their ER values: anti-substrates (ER < 1) exhibiting low P-gp affinity and primarily interacting with the OBS, intermediate compounds (1 ≤ ER ≤ 2) displaying moderate P-gp affinity with relatively equal distribution between the MBC and OBS, and strong substrates (ER > 2) demonstrating high P-gp affinity and preferential binding to the MBC for efficient transport. This classification, which derives from in vitro assays where low-ER compounds are less likely to be P-gp substrates and high-ER compounds are actively effluxed, enables the assessment of diverse compound-P-gp interactions and provides insights into transport mechanisms. This study investigates the molecular basis of P-gp substrate recognition by analyzing the binding of 31 compounds with varying ER values to distinct regions of the P-gp protein structure. The drug efflux process begins with the binding of the substrate within or close to specific transmembrane (TM) helices, primarily TM6 and TM12, which form the primary regions of the drug-binding pocket where the substrates are recognized and bound, as well as TM1, TM4, TM10, and TM11, which contribute to shaping the drug-binding pocket and influencing the specificity and size of substrates that can be accommodated by P-gp. During the transport cycle, P-gp switches among different conformations that are vital for substrate translocation. When at rest, P-gp adopts a “closed” configuration with the two nucleotide-binding domains, 1 and 2 (NBD1 and NBD2), in proximity. Upon interaction with a substrate molecule in the drug-binding pocket, a conformational shift occurs, initiating a transporting cycle facilitated by specific amino acids within the transmembrane domains [12].

While the exact mechanism of P-gp-mediated transport remains unknown, three primary models have been proposed: direct extraction, flippase activity, and membrane extraction. These models, though not mutually exclusive, offer different perspectives on how P-gp interacts with substrates and facilitates their efflux (Figure 2) [16].

The direct extraction model suggests that P-gp directly extracts substrates from the cytoplasm, like ion-transporting ATPases. While this model provides a simplified explanation, it fails to fully account for the broad substrate specificity and membrane interactions of P-gp (Figure 2(1)). The flippase model, supported by experimental evidence [19], proposes that P-gp acts as a lipid flippase, transporting substrates from the inner to the outer leaflet of the plasma membrane. This mechanism requires specific substrate recognition and energy from ATP hydrolysis (Figure 2(2)). The membrane extraction model suggests that P-gp directly removes lipophilic substrates from the outer leaflet of the membrane, preventing their cellular entry. This model is supported by studies demonstrating the interaction of P-gp with substrates within the membrane and the influence of substrate concentration on transport rates (Figure 2(3)) [12,16].

Although the exact mechanism of P-gp-mediated transport remains elusive, it is likely a combination of these models. The used specific mechanism may vary depending on the substrate, cellular conditions, and the conformational state of the protein [20].

Further research is crucial to elucidate the intricate details of P-gp’s transport cycle and its implications for drug resistance and cellular homeostasis. To overcome P-gp-mediated drug resistance, researchers are exploring several strategies, including the development of P-gp inhibitors and drug delivery systems that can circumvent P-gp activity.

Recent research has significantly advanced our understanding of P-gp structure and function. Cryo-EM studies have revealed four distinct conformational states of human ABCB1 (P-gp) in a lipid environment: the apo state, substrate-bound state, inhibitor-bound state, and nucleotide-trapped state. These structures provide detailed views of the conformational transitions associated with ligand and nucleotide binding, offering a mechanistic framework for how P-gp recognizes and transports diverse substrates. The structural transitions in P-gp are asymmetric, with the C-terminal half remaining more rigid compared to the N-terminal half. TM4 and TM10 undergo the most significant rearrangements during the transport cycle, underscoring their crucial role in substrate translocation and inhibitor binding [21].

The apo state of P-gp, traditionally depicted as a symmetric inward-facing conformation, adopts an asymmetric inward-facing closed conformation, challenging previous models. In this state, transmembrane helix 4 (TM4) plays a crucial auto-inhibitory role by occluding the substrate-binding site, acting as an “affinity gate” to limit substrate access. Substrate and inhibitor binding induce markedly different conformational changes. Substrates like Taxol bind asymmetrically, primarily within the C-terminal domain, while inhibitors like zosuquidar fully occlude the central cavity. This highlights the distinct binding interactions that differentiate substrates and inhibitors, influencing P-gp’s function [21]. Understanding the mechanisms of P-gp-mediated drug resistance is pivotal for developing more effective cancer therapies, particularly those aimed at manipulating the protein’s structure and biochemical behavior. However, this approach necessitates careful consideration, as P-gp is ubiquitously expressed throughout the body. Systemic modifications to its biochemistry can have significant and potentially detrimental consequences for the patient [13].

## 3. ABCB1 Gene Expression

The P-gp encoded by the *ABCB1* gene (also known as *MDR-1*), located on chromosome 7q21.12, is expressed in multiple tissues, including the in liver, kidney, intestine, brain, testes, adrenal gland, uterus, ovary, placenta, and pancreas [22].

The *ABCB1* gene has been found to have two promoters: a proximal (downstream) promoter and a distal (upstream) promoter. Transcripts originating from the upstream promoter consist of 29 exons, whereas those derived from the downstream promoter consists of 28 exons [23]. Current evidence suggests that the 28-exon transcript is predominantly expressed in normal, functioning cells, while drug-resistant cancer cells are more likely to express the 29-exon transcript at higher frequencies.

The expression of P-gp is subject to regulation through several mechanisms, including circadian rhythms, microRNAs, RNA-binding proteins, epigenetic modifications, and signaling pathways [16]. In cancer cells, the P-gp expression is further upregulated through a combination of genetic and epigenetic alterations and, in response to various environmental stressors within the tumor microenvironment [5]. These regulatory processes are elaborated upon in subsequent sections.

The regulation of the ABCB1 gene varies greatly between species. For example, in a study [24] investigated the ABCB1 gene in guinea pigs, revealing a greater degree of isoform diversity compared to human P-gp. The guinea pigs exhibited three distinct ABCB1 mRNA isoforms generated through alternative splicing and exon usage, with varying expression patterns across tissues like placenta and brain micro-vessels. These isoforms displayed differences in their 3′ untranslated regions and C-terminal amino acid sequences, suggesting potential impacts on protein function and regulation. In contrast, human P-gp, while encoded by a single gene, exhibits a limited isoform diversity [24]. While the human ABCB1 exhibits polymorphisms that can lead to amino acid substitutions in the protein, these variations do not necessarily create distinct isoforms in the same way observed in guinea pigs. This example shows the variety of the regulatory mechanisms that the ABCB1 gene can use.

### 3.1. ABCB1 Regulation

#### 3.1.1. Circadian Regulation

Adenosine deaminase acting on RNA 1 (ADAR1), a crucial enzyme facilitating adenosine-to-inosine (A-to-I) RNA editing, plays a significant role in modulating the circadian expression of the ABCB1 gene [25]. This post-transcriptional modification by ADAR1 influences gene expression and protein function, impacting cellular physiology. Notably, studies conducted on renal proximal tubule epithelial cells (RPTECs) have demonstrated a direct link between ADAR1 activity and the 24 h oscillation of P-gp levels, indicating a circadian regulatory mechanism. While ADAR1 does not directly edit the ABCB1 transcripts, its downregulation induces alternative splicing within intron 27 of the ABCB1 gene. This splicing alteration leads to the production of retained intron transcripts that are susceptible to nonsense-mediated mRNA decay, ultimately destabilizing ABCB1 mRNA. Consequently, the disruption of this splicing mechanism impairs the 24 h oscillation of P-gp expression, thereby affecting the time-dependent renal elimination of P-gp substrates [26].

Notably, research on the blood–brain barrier further elucidates the systemic importance of circadian P-gp (ABCB1) activity. Studies reveal that xenobiotic efflux follows a circadian pattern, peaking during the active phase, mirroring the time-dependent variations seen in renal elimination. This circadian modulation is mediated via intracellular magnesium levels, regulated by the molecular clock through transient receptor potential melastatin-subfamily member 7, directly influencing P-gp’s efflux efficiency [27].

Therefore, understanding the interconnected nature of P-gp regulation across tissues, including renal cells and the blood–brain barrier, provides a comprehensive view for therapeutic optimization. Recognizing the circadian rhythm of P-gp expression is crucial for optimizing chronotherapy in hematological malignancies, especially those with central nervous system involvement. Modulating ADAR1 activity or targeting splicing, alongside considering these circadian rhythms, could enhance chemotherapeutic efficacy by increasing intracellular drug accumulation and optimizing administration timing, ultimately improving patient outcomes.

#### 3.1.2. Post-Transcriptional Regulation

MicroRNAs (miRNAs), approximately 22 nucleotides, regulate gene expression post-transcriptionally by binding to the 3′ untranslated region (UTR) of target mRNAs, leading to translational repression or degradation [28,29].

This binding, crucial for regulating drug transporters and other key proteins, is primarily determined by the “seed region,” nucleotides 2-7/2-8 at the miRNA’s 5′ end. While seed region complementarity to the 3′ UTR is pivotal, additional pairing enhances target specificity and stability. Consequently, miRNAs are recognized as key regulators in several biological pathways, including the modulation of P-gp expression. Specifically, miRNAs such as miR-27a, miR-298, and miR-451 regulate P-gp expression by directly targeting its mRNA [28]. While miRNA–mRNA binding often involves this seed region, non-canonical interactions also contribute to the complexity of this regulatory mechanism [29].

Several miRNAs play a significant role in the post-transcriptional regulation of P-gp; below, we show some of the most recent studies related to hematological cancers.

While the aforementioned miRNAs play significant roles in P-gp regulation in several cancers, their implications in hematological malignancies are also being explored. In these malignancies, miRNAs are crucial in influencing drug resistance and tumor progression. For example, miR-142, highly expressed in hematopoietic cells, is implicated in lymphomas and leukemias, and its dysregulation can potentially influence drug resistance mechanisms [30]. Specifically, miR-142′s role in regulating crucial signaling pathways within hematopoietic cells may indirectly affect P-gp expression by altering the activity of transcription factors that control MDR1 gene expression. Furthermore, miRNAs within extracellular vesicles have been shown to transfer drug resistance traits between cells, underscoring their systemic impact on tumor microenvironments [31]. The complex interplay between miRNAs and transcription factors like NF-κB, which directly binds to the MDR1 promoter, highlights the intricate regulatory network controlling P-gp expression. Although specific studies directly linking miRNAs to P-gp regulation in hematological malignancies are limited, insights can be drawn from mechanisms observed in other cancers. For instance, miR-129 and miR-29a have been shown to directly target P-gp, downregulating its expression and reversing drug resistance [32,33]. Similarly, miR-146a-5p regulates the TRAF6/NF-kB p65/P-gp axis in pancreatic cancer, affecting chemoresistance, which could be a potential target in hematological malignancies [34]. The TRAF6/NF-κB axis, involving TRAF6, a key component in Toll-like receptor and interleukin-1 receptor signaling, ultimately leads to NF-κB p65 phosphorylation and binding to the MDR1 promoter.

The complex regulation of P-gp expression involves a multitude of factors, including transcriptional and post-transcriptional mechanisms. A deeper understanding of these regulatory pathways is crucial for developing strategies to modulate P-gp activity and overcome drug resistance in several diseases, particularly cancer. By targeting specific regulatory mechanisms, such as miRNA-mediated inhibition or pharmacological modulation of signaling pathways, it may be possible to enhance drug efficacy and improve patient outcomes.

In conclusion, miRNAs are pivotal in regulating drug resistance in hematological malignancies, with potential implications for P-gp regulation. While direct evidence in hematological contexts is sparse, the established roles of miRNAs in other cancers provide a foundation for further research. Understanding these interactions may lead to novel therapeutic strategies to combat drug resistance in hematological malignancies.

#### 3.1.3. Epigenetic Regulation

Epigenetic mechanisms, including DNA methylation and histone modifications, have been implicated in the regulation of *ABCB1* expression.

Hypermethylation of the downstream *ABCB1* promoter has been linked to increased utilization of the upstream promoter, leading to higher *ABCB1* expression [23]. This epigenetic alteration has been observed in cancer cell lines resistant to chemotherapies such as cisplatin, docetaxel, and doxorubicin [16].

In addition to DNA methylation, histone modifications also play a significant role in regulating the *ABCB1* expression. Inhibition of histone deacetylases (HDACs), particularly HDAC1 and HDAC2, has been shown to upregulate P-gp expression in human placental trophoblast cells [35]. This upregulation is mediated through increased histone acetylation, which promotes *ABCB1* gene transcription. A negative linear relationship between HDAC1/2 mRNA and *ABCB1* mRNA expression further supports the inhibitory role of these HDACs on P-gp expression.

A study examined the epigenetic mechanisms underlying *MDR1* gene downregulation in prostate cancer [23]. The authors found that increased promoter methylation levels were associated with decreased P-gp expression in prostate cancer compared to normal prostate tissues and high-grade prostatic intraepithelial neoplasia (HGPIN). However, treatment with a DNA methyltransferase inhibitor alone had limited effects on *MDR-1* expression. In contrast, treatment with a histone deacetylase inhibitor, trichostatin A (TSA), either alone or in combination with a DNA methyltransferase inhibitor, led to a more robust re-expression of *MDR1*. This suggests that histone modifications, particularly histone acetylation, play a more dominant role in regulating *MDR1* expression in prostate cancer [23].

These findings collectively highlight the complex interplay between DNA methylation and histone modifications in regulating *ABCB1* expression. Targeting these epigenetic mechanisms may offer novel therapeutic strategies to overcome drug resistance in cancer.

#### 3.1.4. Signaling Pathways

Intracellular acidification, a common feature of the tumor microenvironment, has been shown to significantly influence P-gp expression by modulating specific MAPK signaling pathways. Acidification downregulates P-gp expression by inhibiting the p38 MAPK pathway and activating the JNK pathway [36,37]. This complex interplay between these two signaling cascades suggests a fine-tuned mechanism by which cells respond to acidic stress.

The *MDR1* gene promoter contains a negative binding site for the AP-1 heterodimeric transcription factor, particularly the c-Jun/c-Fos dimer [37]. LLA studies have revealed that intracellular acidification leads to the upregulation of AP-1 components, c-Jun and c-Fos, in drug-resistant cancer cells such as K562/DOX. This specific cell line created by inducing drug resistance in the K562 leukemia cell line by exposing it to the chemotherapeutic drug doxorubicin serves as a model for studying multidrug resistance mechanisms, particularly related to P-gp expression and regulation, regarding leukemia treatment. The K562/DOX cells exhibit resistance to doxorubicin and overexpress P-gp [36]. This suggests a potential link between acidification and the regulation of *MDR1* gene expression through AP-1 transcriptional activity. Further investigation into the specific molecular mechanisms underlying AP-1-mediated regulation of drug resistance is warranted.

Another transcription factor involved is Yin Yang-1 (YY1). It plays a crucial role in regulating the expression of the multidrug resistance gene *MDR1* in acute lymphoblastic leukemia (ALL). YY1 binds to multiple sites on the *MDR1* promoter, directly regulating its transcription. Silencing YY1 in ALL cells leads to decreased MDR1 expression and increased drug sensitivity, confirming its role in drug resistance. Clinical analysis has shown that high YY1 expression correlates with decreased overall survival in ALL patients, suggesting its potential as a prognostic marker and therapeutic target [38]. Beyond MDR1, YY1 regulates genes involved in cell growth, proliferation, and apoptosis, highlighting its broader significance in tumorigenesis. In addition, it can act as both an activator and repressor of transcription, depending on the context, interacting partners, and chromatin structure [39]. Its association with chemoresistance underscores its potential as a therapeutic target to overcome this major clinical challenge in cancer treatment.

CD133, a cell surface marker associated with cancer stem cells, has been implicated in the regulation of P-gp expression. CD133 overexpression promotes chemoresistance, while CD133 knockdown enhances drug sensitivity in gastric cancer cells. CD133 interacts with PI3K-p85, a regulatory subunit of PI3K, influencing PI3K enzymatic activity and downstream AKT signaling. Song et al., 2018, also demonstrated that this interaction is caried out through the tyrosine residue 852 of CD133 and promotes mRNA expression of *MDR1* gene, contributing to the enhanced expression of P-gp and multidrug resistance [40]. Targeting the CD133-PI3K-p85 interaction may offer a novel therapeutic strategy for overcoming drug resistance in gastric cancer.

As previously discussed, P-gp exhibits widespread tissue distribution. Notably, it is highly expressed in the intestinal epithelium. Evidence suggests a strong link between P-gp dysfunction and increased susceptibility to intestinal inflammation. Until now, several signaling pathways regulate P-gp expression in the intestinal epithelium, including Wnt, transforming growth factor beta (TGF-β), and mitogen-activated protein kinase (MAPK) pathways. These pathways modulate P-gp transcription in response to various stressors and stimuli. Furthermore, accumulating evidence indicates that microbial metabolites, such as butyrate and secondary bile acids, can induce P-gp expression in the intestine. This induction likely involves a complex signaling network encompassing histone deacetylase inhibition and the activation of nuclear receptors, such as hepatocyte nuclear factor 4 alpha (HNF4α) [41].

In summary, signaling pathways, including MAPK, AP-1, and PI3K/AKT, as well as transcription factors like YY1, regulate P-gp expression. A comprehensive understanding of these regulatory mechanisms is essential for developing effective strategies to overcome drug resistance and improve cancer therapy.

### 3.2. MDR1 Polymorphisms

Over 50 single nucleotide polymorphisms (SNPs) of the *MDR1* gene have been identified, with some known to affect the function of P-gp encoded by the gene. *MDR1* gene polymorphism can influence the expression and function of P-gp and have been associated with conditions such as inflammatory bowel disease, lung cancer, and renal epithelial tumors. However, the role of these polymorphisms in the development of autoimmune diseases remains unclear [4]. Herein, we will scrutinize some of the most salient ones related to hematological pathologies, focusing particularly on their impact in Chronic Myeloid Leukemia (CML), Acute Myeloid Leukemia (AML), and Multiple Myeloma.

The first reported polymorphism was the G2677T variant, resulting in amino A893S acid substitution. This polymorphism, along with the 1236C>T variant, has been implicated in AML. A meta-analysis of observational studies in AML patients [42] found that these polymorphisms can influence the effectiveness of standard treatment, with some associated with improved overall survival, providing clinical evidence of their relevance [42].

The SNP 3435 C>T has been particularly well-studied. It has been associated with ulcerative colitis and reduced P-gp expression in the intestine [6]. However, it is also relevant in myeloma patients. The study [41], highlighted a significant finding regarding the MDR1/3435 (C>T) polymorphism, particularly in patients treated with a combination of PLD (pegylated liposomal doxorubicin) and bortezomib. Patients with myeloma carrying this polymorphism, specifically the T/T allele, are associated with weaker drug efflux activity [43]. This means that myeloma cells carrying the T/T allele may experience greater cytotoxicity from drugs like PLD, leading to longer drug exposure and potentially better treatment response. Critically, these patients exhibited better progression-free survival, response rate, and time to progression compared to those with other genotypes, providing concrete clinical evidence for the impact of this polymorphism on patient outcomes in MM treated with this regimen. In contrast, no such correlation was observed between this genetic variant and treatment outcomes in patients receiving bortezomib alone. PLD is important because it allows for better drug delivery and reduces toxicity compared to standard doxorubicin; it encapsulates doxorubicin in liposomes, allowing for longer circulation time, enhanced tumor uptake, and reduced side effects, making it an effective treatment option for several cancers, including myeloma [44]. This disparity highlights the potential impact of individual genetic variations on the effectiveness of particular drug combinations in patients with advanced multiple myeloma. Notably, this polymorphism may have the potential to serve as a valuable biomarker for optimizing treatment outcomes in these patients.

Imatinib mesylate, a primary treatment for chronic myeloid leukemia (CML), targets the BCR-ABL oncogene. However, patient responses vary, prompting investigations into the pharmacogenetics of imatinib metabolism, particularly concerning ABCB1/MDR1 gene polymorphisms. A systematic review and meta-analysis of 12 clinical studies, encompassing 1826 CML patients [45], examined the impact of ABCB1 polymorphisms on imatinib response. This comprehensive clinical analysis revealed that the c.2677G and c.3435T alleles correlate with reduced therapeutic efficacy, whereas the c.1236CC genotype, especially in Asian patients, is associated with improved outcomes. Specifically, the c.3435C>T polymorphism significantly influenced imatinib response, with the 3435T allele predicting poorer outcomes (OR = 0.85, *p* = 0.042). These findings suggest that ABCB1 gene variations significantly impact imatinib pharmacokinetics, contributing to inter-patient variability in treatment responses. The data underscore the potential of genetic profiling to personalize CML therapy, emphasizing the need for further research to elucidate the underlying mechanisms and clinical implications of these genetic variations.

In conclusion, findings from these concrete clinical studies and meta-analyses highlight the potential for personalized medicine through genetic profiling. Further research into the mechanisms and clinical implications of these polymorphisms is crucial for optimizing therapeutic strategies and improving patient outcomes in these diseases.

## 4. Importance of P-Glycoprotein in Hematological Malignancies

Overexpression of P-gp is a well-established mechanism of chemoresistance in hematological cancers, including AML, ALL, diffuse large B-cell lymphoma, multiple myeloma, and follicular lymphoma. In these malignancies, increased P-gp expression in malignant hematopoietic cells is often associated with the activation of signaling pathways that promote drug resistance, such as the PI3K/Akt pathway. This enhanced efflux capacity reduces intracellular drug concentrations, hindering the effectiveness of chemotherapy. While P-gp poses a significant challenge, it is important to note that not all therapies are affected; for instance, monoclonal antibodies targeting cell surface markers such as CD20 (used in lymphomas) or CD38 (used in multiple myeloma) are not P-gp substrates and, thus, their efficacy is not directly compromised by P-gp overexpression. Therefore, understanding the molecular mechanisms driving P-gp overexpression and its contribution to drug resistance is essential for developing strategies to improve treatment outcomes in hematological malignancies. Targeting P-gp through inhibitors, or circumventing its efflux function through alternative therapeutic approaches, holds considerable promise for overcoming multidrug resistance and enhancing the effectiveness of cancer therapies.

In this section, we will discuss the role of P-gp in the most common hematological malignancies and its relation with its treatment [7].

### 4.1. Multiple Myeloma

Multiple myeloma (MM) is a systemic malignancy characterized by the uncontrolled proliferation of clonal plasma cells in bone marrow that leads to the overproduction of monoclonal immunoglobulin proteins or their fragments, which can accumulate in the blood and urine [46]. MM accounts for approximately 1% of all cancers worldwide and 10–15% of all hematological neoplasms. While it remains an incurable disease, significant therapeutic advancements, including proteasome inhibitors and immunomodulatory drugs, have considerably improved patient outcomes. High-dose chemotherapy with autologous stem cell transplantation remains a cornerstone of treatment for eligible patients. Supportive care, including the management of treatment-related side effects and myeloma-associated organ damage, is essential for optimizing patient quality of life and overall survival [46,47].

MM is a challenging disease to treat due to the development of drug resistance, where efflux transporters, such as P-gp, play a crucial role. P-gp overexpression has been observed in a high percentage of patients following treatment with specific drug combinations commonly used in MM therapy, especially with regimens like doxorubicin/vincristine and VAD (90–100%), clinically hindering drug efficacy and contributing to treatment failure [47]. Although P-gp gene expression increases with the progression of the disease in MM patients, data from patient cohorts indicate that higher P-gp expression does not significantly affect the survival of newly diagnosed MM patients treated with bortezomib [48]. While large prospective clinical trials designed solely to link P-gp expression to overall MM outcomes independent of specific drug regimens are less common, numerous studies in patient cohorts treated with standard therapies provide clinical evidence suggesting P-gp’s involvement in resistance and its potential impact on prognosis depending on the specific drugs used [49].

Using the CDy1 efflux assay, it has been reported that a subpopulation of MM cells overexpressing membrane P-gp and mRNA showed resistance to carfilzomib, a second-generation proteasome inhibitor approved for therapy-refractory MM. The resistance to carfilzomib correlated with increased transporter activity as measured by CDy1 efflux [50]. Additionally, studies have analyzed the effects of upregulated *ABCB1* expression on resistance to carfilzomib in MM cell lines, such as RPMI-8226/Dox40 and KMS-34/Cfx. The findings indicate that increased P-gp expression conferred resistance to carfilzomib, with cotreatment with vismodegib reversing this resistance. This finding, in a clinically relevant context, highlights P-gp’s role in resistance to newer proteasome inhibitors [50].

However, studies have shown that targeting P-gp with specific inhibitors may not always translate into significant clinical benefits. For instance, Mynott et al., 2021, demonstrated that the inhibition of P-gp using the specific inhibitor tariquidar glucosylceramide synthase did not enhance the efficacy of bortezomib or carfilzomib in MM cell lines, except at a high, clinically unachievable concentration [48]. These findings suggest that targeting P-gp alone may not be sufficient to overcome drug resistance in MM, highlighting the complex interplay of resistance mechanisms in this malignancy.

### 4.2. Leukemias

Leukemias are a diverse group of cancers originating in the blood and bone marrow, characterized by the uncontrolled proliferation of abnormal blood cells. Classification is based on cell lineage and disease progression. Modern classifications, such as the International Consensus Classification (ICC), refine the distinction between main leukemia types—acute lymphoblastic leukemia (ALL), acute myeloid leukemia (AML), chronic lymphocytic leukemia (CLL), and chronic myeloid leukemia (CML)—which are uniquely linked to the Philadelphia chromosome (BCR::ABL1 fusion gene) [51,52,53,54]. These classifications integrate morphology, clinical data, and genomics, improving diagnosis and patient outcomes. For instance, in Chronic Myeloid Leukemia (CML), tyrosine kinase inhibitors (TKIs) like imatinib, nilotinib, and dasatinib represent a first-line non-chemotherapeutic approach, yet resistance to these agents, often involving P-gp, remains a clinical challenge [55,56]. Similarly, in Acute Lymphoblastic Leukemia (ALL), while chemotherapy remains central, targeted therapies such as tyrosine kinase inhibitors (e.g., for Ph+ ALL) and monoclonal antibodies can also be affected by MDR. Understanding the interplay between these modern targeted therapies and P-gp-mediated resistance is crucial for improving patient outcomes in leukemia.

P-gp overexpression is frequently observed in leukemia cells and is a significant contributor to drug resistance [56]. By actively effluxing chemotherapeutic agents, P-gp reduces intracellular drug concentrations and diminishes therapeutic efficacy. This mechanism of drug resistance is particularly relevant in acute leukemias, such as ALL and AML, where P-gp overexpression has clinically associated with poor treatment outcomes in patients [55,57]. Specifically, studies in AML patients have consistently demonstrated a correlation between high P-gp expression and lower rates of complete remission and poorer survival outcomes [58].

Understanding the underlying signaling pathways, including MAPK, NF-κB, Wnt/β-catenin, and PI3K/Akt, is crucial to counteract the P-gp-mediated resistance [59,60,61]. Targeting these signaling pathways may offer potential strategies to modulate P-gp expression and enhance the efficacy of chemotherapy in leukemia. However, as discussed earlier, strategies targeting P-gp directly, such as the use of P-gp inhibitors, have shown limited success in clinical trials.

The co-expression of P-gp with other proteins, such as nestin, has been observed in leukemia cell lines. Nestin, a type VI intermediate filament protein, is typically expressed in neural stem cells, but has also been found in cancer cells, including leukemia. The co-expression of P-gp and nestin suggests a potential functional relationship between these two proteins in promoting drug resistance. Notably, the co-expression of nestin and P-gp has been reported in both human neural stem/progenitor cells and multidrug resistance variants of leukemia cells, indicating a potential role for nestin in regulating P-gp expression and function [62].

Acute myeloid leukemia (AML), the most prevalent acute leukemia in adults, affects approximately 3.4 individuals per 100,000 annually, disproportionately impacting the elderly [63]. Despite advancements in treatment, the 5-year overall survival rate remains lower in young adults compared to pediatric patients. The standard “7 + 3” (a common treatment protocol used for AML that involves a combination of Cytarabine and Daunorubicin) chemotherapy regimen exhibits limited efficacy, resulting in a complete remission rate of approximately 40% and a median overall survival of 12–18 months [64]. A significant contributor to this therapeutic challenge is the overexpression of ATP-binding cassette (ABC) transporters, particularly P-gp, in AML cells, which leads to treatment resistance in approximately 40% of patients. Examples of chemotherapeutic drugs commonly used in hematological malignancies that are P-gp substrates include anthracyclines such as doxorubicin [17], vinca alkaloids such as vincristine [65], and tyrosine kinase inhibitors such as imatinib [66,67].

In resistant AML cells, the overexpression of acid ceramidase (AC) has been demonstrated to contribute upregulation of P-gp. Conversely, pharmacological or genetic inhibition of AC results in a significant reduction in P-gp protein levels, thereby restoring cellular sensitivity to chemotherapeutic agents. Mechanistically, the NF-κB signaling pathway plays a central role in AC-mediated P-gp modulation. AC overexpression induces the activation of NF-κB, leading to increased transcription of P-gp. This observation is corroborated by studies showing that sphingosine kinase inhibitors and NF-κB inhibitors attenuate P-gp expression in AML cells, further substantiating the involvement of these pathways in AC-mediated drug resistance [68].

Furthermore, clinical investigations involving AML patient samples have revealed a significant positive correlation between PKCε protein expression and P-gp protein levels. PKCε, an isoform of the protein kinase C family, exhibits frequent overexpression in AML and is associated with adverse clinical outcomes. This isoform appears to drive treatment resistance by enhancing P-gp expression. However, it is noteworthy that PKCε overexpression does not confer resistance to cytarabine, a chemotherapeutic agent that is not a substrate for P-gp. This specificity highlights that the impact of PKCε on drug resistance, exemplified by resistance to daunorubicin (DNR), is contingent upon the drug’s susceptibility to P-gp-mediated efflux [49]. Consequently, the selective upregulation of P-gp by PKCε underscores the importance of considering substrate specificity in the context of P-gp-mediated drug resistance in AML. A study by Prijić et al., 2015, in a cohort of 118 AML patients showed a significant correlation between P-gp expression/activity and patient age, white blood cell count, and red blood cell count, impacting overall survival [69]. Higher P-gp activity predicted poorer overall survival, particularly in AML with myelodysplasia-related changes. The study also identified a positive correlation between Akt and p38 phosphorylation, suggesting co-activation. Intriguingly, a negative correlation between P-gp activity and p38 phosphorylation was observed, indicating a potential negative regulatory effect of MAPK on P-gp, possibly contributing to resistance. These findings suggest that P-gp activity, along with phosphorylated Akt and ERK1/2, could be independent prognostic markers in AML.

Finally, P-gp overexpression on lymphocytes has been significantly correlated with failed molecular response to imatinib in chronic myeloid leukemia patients [70]. This key clinical observation suggests that P-gp expression levels may serve as a diagnostic marker for multidrug resistance in leukemia, aiding in the assessment of treatment response and disease progression. Importantly, increased P-gp activity has been clinically associated with poor prognosis and disease relapse, emphasizing the need for monitoring P-gp levels during treatment. This underscores the critical role of P-gp in influencing treatment outcomes and highlights the importance of developing strategies to overcome P-gp-mediated drug resistance.

ABCB1 overexpression is a pivotal mechanism of resistance to tyrosine kinase inhibitors (TKIs) in Chronic Myeloid Leukemia (CML), impacting drugs such as imatinib, nilotinib, and dasatinib. Clinical studies demonstrate that this elevated expression, observed early in resistance development, reduces intracellular TKI concentrations, contributing to poorer patient responses [71]. Furthermore, genetic polymorphisms within ABCB1, including 1236C>T, 2677G>T, and 3435C>T, modulate TKI response and intracellular drug accumulation, as shown by clinical analyses and meta-analyses [45,72,73]. Notably, high ABCB1 expression correlates with diminished responses to imatinib and serves as a predictor for resistance development, including kinase domain mutations [74]. The regulation of ABCB1 expression, influenced by epigenetic factors and transcriptional pathways like Wnt/β-catenin, further complicates treatment outcomes, as early increases in ABCB1 expression are associated with poorer event-free survival. While ABCB1 expression is generally elevated in CML, its predictive value for relapse following TKI discontinuation is limited, as evidenced by the observed downregulation of ABCB1 in relapsed patients upon TKI withdrawal [75,76]. Consequently, monitoring ABCB1 expression during active treatment remains crucial for tailoring therapeutic strategies, although its utility as a relapse predictor is less established.

In Acute lymphoblastic leukemia (ALL), this role of P-gp extends beyond mere drug efflux, influencing overall prognosis. Specifically, polymorphisms within the MDR1 gene, which encodes P-gp, have been identified as potential markers for risk stratification. For instance, the C3435T and C1236T polymorphisms are significantly associated with high-risk groups in childhood ALL, suggesting their utility in identifying patients requiring more intensive therapeutic strategies [77,78]. A meta-analysis of clinical studies has shown that the association of the C3435T polymorphism with ALL risk is predominantly observed in Asian populations, highlighting potential ethnic variations in genetic predispositions [79].

Furthermore, high P-gp expression levels are consistently linked to poor prognosis and reduced survival rates in ALL patients [56]. This overexpression contributes to multidrug resistance, a major obstacle in ALL treatment. Consequently, genetic variations in MDR1, which alter P-gp function, have a direct impact on the pharmacokinetics of anticancer drugs, thereby influencing treatment efficacy [80].

Beyond the influence of MDR1 polymorphisms, the expression levels of the MDR1 gene itself have clinical significance in pediatric ALL. While a study found no overall association between MDR1 expression and clinical parameters or treatment response, they did observe that 21% of MDR1-positive cases exhibited strong expression. This suggests that, in a subset of patients, high MDR1 expression may indeed impact treatment outcomes, potentially contributing to drug resistance. Therefore, while not a universal predictor, the intensity of MDR1 expression warrants consideration in the context of personalized treatment strategies for pediatric ALL [81].

Moreover, polymorphisms in the ABCB1 gene, encoding P-gp, have been shown to influence both prognosis and toxicity in pediatric ALL. Specifically, the 1199GA variant is associated with a significantly increased relapse risk, particularly in high-risk patients, whereas the 3435TT and 3435CT variants correlate with a reduced relapse risk compared to the 3435CC genotype. Additionally, the 3435TT variant correlates with reduced bone marrow toxicity during induction therapy, while the 3435CC genotype is associated with increased liver toxicity following high-dose methotrexate treatments [82]. These findings emphasize the importance of incorporating ABCB1 polymorphism analysis into risk stratification and personalized treatment strategies for pediatric ALL.

P-gp overexpression is a critical factor in leukemia drug resistance, particularly in AML and ALL, impacting treatment outcomes. Genetic variations in MDR1, like specific polymorphisms, serve as potential markers for risk stratification and personalized therapy. Beyond drug efflux, P-gp’s interaction with signaling pathways and proteins like AC, PKCε, and LRP underscores the complexity of resistance. While direct P-gp inhibition has been challenging, targeting its regulatory mechanisms offers promising therapeutic avenues. Modulating pathways like NF-κB and PKCε could restore chemosensitivity, and genetic profiling enables tailored drug selection to minimize toxicity. Monitoring LRP in pediatric ALL, along with P-gp, can further refine treatment strategies.

Leveraging our understanding of P-gp’s regulatory mechanisms, genetic variations, and interactions with other proteins is crucial for developing effective leukemia therapies. Future research should prioritize validating these approaches and translating them into clinical practice. By focusing on personalized medicine, modulating key signaling pathways, and refining risk stratification through monitoring relevant proteins, we can significantly improve treatment outcomes and enhance patient survival in leukemia.

### 4.3. Lymphomas

Lymphomas, a diverse group of blood cancers originating in the lymphatic system, are primarily classified based on the lymphocyte type involved: B-cell, T-cell, or NK-cell. The World Health Organization (WHO) classification is the most widely accepted framework, considering morphology, immunophenotype, genetics, and clinical presentation. B-cell lymphomas, the most common type, include diffuse large B-cell lymphoma, follicular lymphoma, mantle cell lymphoma, and marginal zone lymphoma [83]. T-cell and NK-cell lymphomas, such as peripheral T-cell lymphomas and anaplastic large-cell lymphoma, are less common and often more aggressive. Hodgkin lymphoma, distinct from non-Hodgkin lymphomas, is characterized by the presence of Reed–Sternberg cells. Previous classification systems, such as the Revised European-American Lymphoma (REAL) classification and the Kiel classification, contributed to the development of the current WHO classification, emphasizing histological and immunophenotypic features [84,85].

About 30% of diffuse large B-cell lymphoma patients display early relapse and 10% show therapy-refractory disease, often due to the development of multidrug resistance. The mechanisms underlying multidrug resistance in lymphomas are complex and include target gene mutations, metabolic reprogramming, overexpression of drug efflux transporters such as P-gp, upregulation of anti-apoptotic proteins, and enhanced DNA damage repair. P-gp increases resistance to chemotherapy, targeted therapy, and immunotherapy in lymphomas, clinically leading to poor treatment outcomes [86].

Non-Hodgkin lymphoma (NHL) is a prevalent hematological malignancy arising from lymphoid tissue and lymph nodes throughout the body. Consequently, NHL can manifest in several anatomical locations. Chemotherapy constitutes the primary therapeutic modality for most NHL subtypes [87].

A clinical study demonstrated the potential use of peripheral bloodCD56+ cells as a surrogate marker for monitoring chemoresistance in NHL patients. This study found that, in chemoresistant NHL patients, the mRNA expression of the *MDR1* gene was significantly elevated in CD56+ cells compared to chemosensitive patients and healthy controls. Additionally, the function of P-gp, as measured by rhodamine (Rho)123 accumulation, was higher in the chemoresistant group. These findings provide clinical evidence suggesting that monitoring P-gp expression and function in peripheral blood CD56+ cells could provide a non-invasive method to assess chemoresistance in NHL patients and guide personalized treatment [87].

Epstein–Barr virus (EBV), a human gamma herpes virus with three major latencies, has been linked to several tumors such as lymphoma and gastric carcinoma. Extranodal NK/T-cell lymphoma (ENKTCL), mostly EBV-positive cases, poses a grim prognosis with characteristics such as low survival rates, metastasis, inflammation, and drug resistance. Poor prognostic molecules and abnormal cells signaling pathways are associated with ENKTCL [88]. EBV infection prompts phosphorylation in NF-κB, MAPK, and JAK/STAT pathways, leading to tumor proliferation and immune suppression. ENKTCL patients often exhibit mutations in tumor suppressor genes. The resistance of ENKTCL to existing anticancer drugs due to P-gp overexpression requires tailored treatment regimens. High levels of reactive oxygen species, linked to EBV, impact prognosis through cellular deregulation and altered signaling pathways [5,89,90].

The research highlighted the involvement of several signaling pathways, such as NF-κB, STAT, and MAPK, in the regulation of P-gp expression and multidrug resistance. The findings suggest that targeting intracellular reactive oxygen species through NecroX-5 could be a promising strategy to overcome drug resistance in EBV-positive cancers, particularly in NK cell lymphoma. Overall, the study underscores the potential of NecroX-5 as a novel therapeutic agent to enhance the efficacy of existing anticancer drugs by mitigating P-gp-mediated multidrug resistance in EBV-related malignancies [88].

The study focused on developing a novel antitumor target for ENKTCL based on the last eight amino acids (aa) of hexokinase domain component 1 (HKDC1) at the C-terminal. The study found that HKDC1 is highly upregulated in ENKTCL and plays a significant role in tumor growth. By delivering a specific peptide (HKC8) based on the last eight aa of HKDC1 at the C-terminal, the study demonstrates the inhibition of HKDC1 association with vascular endothelial growth factor 1 (VDAC1), leading to mitochondrial dysfunction, reactive oxygen species generation, suppression of EBV replication, and P-gp expression. The peptide was effective in suppressing tumor growth in mouse models by inducing apoptosis, DNA damage in EBV-positive ENKTL cells, and inhibiting P-gp expression. The results suggest that HKDC1-based peptides, especially HKC8, could serve as a novel therapeutic target for ENKTCL antitumor drug development, specifically targeting EBV-positive ENKTCL. The study highlights the potential of HKC8 in inhibiting ENKTCL tumor growth through modulation of mitochondrial function and EBV suppression [91].

## 5. P-Glycoprotein Expression in Immune Cell Subsets

P-gp is expressed in tumor cells and in various immune cell subsets, including macrophages, neutrophils, and mononuclear cells such as, for example, dendritic cells, natural killer (NK) cells, and T cells, suggesting a multifaceted role in the modulation of both pro- and anti-tumor immune responses [6,92,93,94]. Clinically, the expression of P-gp on these immune cells has significant relevance for the efficacy of immunotherapies. For instance, the upregulation of P-gp in tumor cells by the PD-1/PD-L1 axis [95] can contribute to resistance against chemotherapeutic agents often used in combination with immune checkpoint inhibitors (ICIs) like anti-PD-1 or anti-PD-L1 antibodies [96]. Furthermore, P-gp expression on specific immune cell populations can directly impact their function and thus the overall outcome of immunotherapy. In multiple myeloma, the presence of P-gp on circulating monoclonal B cells may not only confer resistance to conventional chemotherapy, but could also affect their interaction with and susceptibility to antibody–drug conjugates or CAR-T cell therapies targeting these malignant B cells [6]. Moreover, in chronic myeloid leukemia (CML), while the EURO-SKI trial sub-analysis focused on P-gp (ABCB1) expression in peripheral blood leukocytes as a predictor for treatment-free remission after TKI discontinuation, the baseline P-gp expression levels on T cells and NK cells might also influence the initial immune response to the leukemia and potentially the long-term success of TKI therapy, even if not directly an ‘immunotherapy’ in the classic sense [75]. Within these subsets, P-gp expression and function can vary significantly, contributing to the complexity of its immunomodulatory effects.

Studies have demonstrated the functional expression of the murine P-gp isoform, mdr1a, in subpopulations of T cells and antigen-presenting cells. Pharmacological blockade of P-gp using antagonists like PSC833 has been shown to inhibit alloimmune T cell activation in vitro in a dose-dependent manner. In vivo, this blockade has a profound impact on allograft survival. In a murine cardiac allotransplantation model, P-gp blockade significantly prolonged cardiac allograft survival to an extent comparable to cyclosporine A monotherapy [97]. This prolonged survival was associated with several immunomodulatory effects, including reduced intragraft expression of the costimulatory molecule CD80 (but not CD86), decreased infiltration of CD3+ and CD8+ T cells, and attenuated production of both Th1 (IFN-γ) and Th2 (IL-4) cytokines by graft-reactive T cells. Notably, combining P-gp blockade with CD86 inhibition resulted in a synergistic effect, leading to even a more significant prolongation of allograft survival and a more potent suppression of IFN-γ and IL-4 production compared to either treatment alone [97]. These findings collectively highlight a novel in vivo regulatory role for P-gp in alloimmunity, acting through the modulation of antigen-presenting cells costimulatory molecule expression and subsequent downstream effects on Th1 and Th2 cytokine production, ultimately suggesting P-gp as a potential therapeutic target for immune regulation in transplantation.

Beyond its role in alloimmunity, P-gp’s influence on anti-tumor immunity is context-dependent. In activated cytotoxic NK cells and specific T cell subsets, P-gp expression is upregulated, potentially enhancing their anti-tumor functions. This suggests that P-gp may be necessary for optimal cytotoxic activity in these immune cells. Conversely, P-gp is highly expressed in pro-tumor M2-like macrophages, highlighting the opposing roles P-gp can play within the tumor microenvironment [6]. While peripheral circulating monocytes exhibit low P-gp expression, anti-inflammatory M2 tissue macrophages infiltrating tumors display elevated levels [98], further emphasizing this dichotomy.

While P-gp’s function in conferring multidrug resistance in cancer cells is well-established, its role in different immune cell subsets such as NK cells has been a subject of not-so-extensive investigation. Studies have consistently demonstrated functional P-gp expression in NK cells, utilizing several assays including flow cytometry with anti-P-gp antibodies, RT-PCR for ABCB1 mRNA detection, and functional efflux assays using fluorescent substrates such as rhodamine 123 (Rh123). These studies have shown that NK cells, both from healthy donors and cell lines, actively efflux these and several conventional substrates, and this efflux can be inhibited by MDR1-reversing agents, for example, cyclosporin A (CsA), PSC833, nicardipine, and AHC-52, confirming functional P-gp activity [99,100,101]. However, important distinctions have been observed in P-gp function and characteristics between normal NK cells and those from aggressive NK cell tumors, as well as compared to the canonical P-gp found in multidrug resistant cancer cell lines.

A key finding across several studies is the link between P-gp function and NK cell-mediated cytotoxicity. The inhibition of P-gp activity by MDR1-reversing agents or anti-P-gp antibodies consistently results in a concentration-dependent decrease in NK cell cytotoxicity against target cells [100,101,102]. This suggests that P-gp plays a crucial role in the cytotoxic process itself, potentially by influencing various steps such as target cell binding, calcium-dependent lytic reactions, and the exocytic release of lytic granules [100,101]. Further mechanistic investigations have suggested that P-gp’s role in NK cell cytotoxicity is likely related to the regulation of lysosomal pH homeostasis within cytotoxic granules, rather than calcium homeostasis [102]. Interestingly, while P-gp is expressed in NK cells from both indolent and aggressive NK cell tumors, aggressive tumors exhibit a reduced susceptibility to P-gp modulators, suggesting distinct functional characteristics or the involvement of other resistance mechanisms [99].

Furthermore, comparative analyses of P-gp in NK cells and multidrug-resistant cell lines have revealed notable differences in substrate specificity and protein structure. Unlike multidrug-resistant cell lines that transport a broad range of substrates, NK cells exhibit a restricted substrate profile, efficiently transporting some substrates like Rh123, but not others like daunorubicin or calcein acetoxymethylester [103]. Additionally, NK cells express smaller molecular weight “mini P-glycoproteins” of between approximately 70 and 80 kDa, instead of the classic 170 kDa form found in multidrug-resistant cell lines, with these mini P-gp also showing differential reactivity with various P-gp antibodies [103]. This highlights the existence of distinct P-gp isoforms in NK cells with potentially unique functions.

The interplay between P-gp expression in different immune cell subsets, including CD4+ T cells, NK cells, and macrophages, underscores the complexity of P-gp’s role in tumor immunology [6]. The mutually exclusive patterns of P-gp expression in pro-tumor and anti-tumor immune cells pose a significant challenge in designing effective P-gp inhibition strategies for cancer therapy. Inhibiting P-gp in pro-tumor immune cells could potentially enhance their tumor-killing capabilities. However, this approach may inadvertently impair the cytotoxic functions of anti-tumor immune cells that also rely on P-gp expression. To address this challenge, a deeper understanding of P-gp’s role in the tumor microenvironment and its interactions with both cancer cells and immune cells is crucial. This knowledge will inform the development of more targeted and effective anticancer therapies [6]. Further investigation in this field is necessary to fully elucidate these complex interactions and develop effective therapeutic strategies. While significant research efforts have focused on developing inhibitors to overcome multidrug resistance, a critical gap in our understanding lies in the diverse roles of P-gp within distinct immune cell subsets.

## 6. Strategies to Modulate P-gp Function

### 6.1. Inhibitors

While numerous P-gp inhibitors have been developed and tested in clinical trials, most have failed to demonstrate significant clinical benefits. This is primarily due to the high doses required to effectively inhibit P-gp, which often lead to severe off-target toxicities. Newer generations of more selective and potent P-gp inhibitors, such as zosuquidar, elacridar, and tariquidar, have demonstrated relatively tolerable safety profiles in clinical trials, as shown in Table 1. However, these agents have still failed to show meaningful improvements in anticancer efficacy when combined with standard chemotherapies. For example, a phase III trial evaluating the addition of zosuquidar to standard induction chemotherapy in elderly AML patients showed no benefit in response rates or overall survival [104]. Alternative strategies, such as the development of P-gp-resistant drug analogs or the utilization of nanoparticle drug formulations, have shown greater promise in overcoming multidrug resistance.

Several attempts have been made to directly block P-gp activity to restore the efficacy of anticancer drugs. This has involved the development of several generations of P-gp inhibitors, ranging from first-generation inhibitors like verapamil to third-generation inhibitors like tariquidar and zosuquidar, as shown in Table 1. Despite promising in vitro results, clinical trials of these P-gp inhibitors have largely been unsuccessful, often due to toxicity concerns, unfavorable pharmacological interactions, and pharmacokinetic challenges [8].

The impact of P-gp on the efficacy of specific drugs used in multiple myeloma (MM) treatment has been extensively studied. For instance, bortezomib, a first-generation proteasome inhibitor, is not typically considered a P-gp substrate. However, recent findings suggest that P-gp inhibition with elacridar can enhance cellular sensitivity to bortezomib, indicating a potential role of P-gp in bortezomib resistance. On the other hand, carfilzomib, a second-generation proteasome inhibitor, is highly selective, but can exhibit resistance in the presence of P-gp overexpression. Clinical trials combining carfilzomib with P-gp inhibitors have shown promise in restoring cellular sensitivity to carfilzomib, although challenges persist due to the high inhibitor concentrations required [47].

Recent studies have investigated the potential of combining cepharanthine (CEP) with glucocorticoids (GCs) to enhance the efficacy of GC therapy in hematological malignancies. CEP, a plant alkaloid, has demonstrated a synergistic effect with GCs in increasing the anti-proliferative effects on immune cells, particularly T cells. One of the proposed mechanisms underlying this synergistic effect is the inhibition of P-gp by CEP, which has been observed in various cell subsets [105,106].

By inhibiting P-gp in CD4+ T cells, CD8+ T cells, and lymphocytes, CEP can increase the intracellular accumulation of GCs in chronic immune thrombocytopenia, thereby amplifying their anti-inflammatory and immunosuppressive effects. Additionally, CEP has been shown to promote the translocation of GC receptors to the nucleus in MOLT-4/DNR cells, further potentiating the therapeutic effects of GCs. MOLT-4/DNR cells are a subline of the MOLT-4 cell line, which is originally derived from human T-lymphoblastic leukemia. The “DNR” designation in MOLT-4/DNR indicates that these cells have been exposed to daunorubicin over time, resulting in increased ABCB1 levels [105].

These findings suggest that the combination of CEP and GCs may be a promising therapeutic approach for chronic immune thrombocytopenia, offering a novel strategy to overcome drug resistance and improve treatment outcomes. Different compounds have been used to reduce or inhibit the expression and/or activity of P-gp and correspond to four generations of development (Table 1) [104]. Pre-clinical studies are significantly impacting the design and potential clinical use of P-gp inhibitors. For instance, the identification of WS-716, a potent and specific inhibitor, demonstrated its ability to reverse P-gp-mediated resistance to paclitaxel in various cancer cell lines without altering P-gp expression or significantly interacting with CYP3A4. In vivo studies showed that WS-716 enhanced paclitaxel sensitivity in MDR tumor models, including patient-derived xenografts, without notable adverse reactions [107]. Furthermore, research into natural P-gp inhibitors has highlighted their potential for effective drug delivery with reduced toxicity [108], while dual inhibitors targeting both P-gp and BCRP have shown promise in improving oral bioavailability and tumor reduction in animal models [109]. The established rhodamine 123 accumulation assay continues to serve as a crucial in vitro tool for predicting clinical P-gp inhibition and potential drug–drug interactions, aiding regulatory and clinical decision-making [110,111]. While the clinical translation of these pre-clinical successes has been historically challenging [7,9], the detailed efficacy and safety profiles of new inhibitors like WS-716 warrant further clinical development [7,107,112]. The authors emphasize that future clinical success will depend on improved patient selection, optimized dosing and combination regimens, and a better understanding of MDR mechanisms and P-gp’s role at physiological barriers [9,12]. Pre-clinical findings are also informing regulatory protocols for managing P-gp-mediated drug–drug interactions in clinical practice [111].

**Table 1:** Clinically relevant P-glycoprotein inhibitors in hematological malignancies.

Author	Inhibitor	Main Results
First generation		
Dalton WS et al., 1995 [113]	Verapamil	No beneficial effect was observed from the addition of oral verapamil to the combination of vincristine, doxorubicin, and dexamethasone chemotherapy regimen for the treatment of drug-resistant myeloma patients.
Lum BL et al., 1992 [114]	Cyclosporine A	High doses of cyclosporine A and etoposide doses should be reduced by approximately 50% to compensate for the pharmacokinetic effects of cyclosporine A on etoposide.
Philip PA et al., 1992 [115]	Nifedipine	Patients with various malignancies received nifedipine at three dose levels; the cardiovascular effects of nifedipine were dose-limiting, but it did not interfere with the pharmacokinetics of etoposide.
Moriki Y et al., 2004 [116]	Pentazocine (PTZ)	The increment of PTZ uptake by the brain could be explained by the saturable efflux transport system involving a P-gp-mediated efflux mechanism of PTZ transport at the blood–brain barrier.
Cunningham C et al., 2009 [117]	Meperidine	Meperidine shows characteristics of an opioid agonist that lacks interaction with P-gp.
Regev R et al., 2007 [118]	Diethyl ether	Anesthetics enhance drug transport across cell membranes, thereby reducing P-gp-mediated multidrug resistance. At high concentrations, this effect is so pronounced that P-gp activity cannot be accurately measured.
Regev R et al.2007 [118]	Chloroform	Anesthetics enhance drug transport across cell membranes, thereby reducing P-gp-mediated multidrug resistance. At high concentrations, this effect is so pronounced that P-gp activity cannot be accurately measured.
Regev R et al., 2007 [118]	Propofol	Anesthetics enhance drug transport across cell membranes, thereby reducing P-gp-mediated multidrug resistance. At high concentrations, this effect is so pronounced that P-gp activity cannot be accurately measured.
Regev R et al., 2007 [118]	Benzyl alcohol	Anesthetics enhance drug transport across cell membranes, thereby reducing P-gp-mediated multidrug resistance. At high concentrations, this effect is so pronounced that P-gp activity cannot be accurately measured.
Gosland MP et al., 1996 [119]	Cefoperazone	Cefoperazone and ceftriaxone showed a remarkable ability to increase the sensitivity of cells resistant to drugs, such as doxorubicin, etoposide, and vinblastine.
Gosland MP et al., 1996 [119]	Ceftriaxone	Ceftriaxone and cefoperazone showed a remarkable ability to increase the sensitivity of cells resistant to drugs, such as doxorubicin, etoposide, and vinblastine.
Fuchs D et al., 2010 [120]	Salinomycin	Salinomycin can reverse multidrug resistance in leukemia stem cell-like cells by inhibiting ABC transporters.
Janneh O et al., 2010 [121]	Nigericin	Increased rh123 accumulation by 1.9-fold.
Asakura E et al., 2004 [122]	Azithromycin	Combination with doxorubicin in K562/ADR cell line: reversed P-gp dependent anticancer drug resistance.
Janneh O et al., 2010 [121]	Brefeldin A	Increase cellular accumulation of zidovudine (P-gp substrate) in the P-gp over-expressing cell line 3T3-F442A.
Janneh O et al., 2010 [121]	Bafilomycin	Increase cellular accumulation of zidovudine (P-gp substrate) in the P-gp over-expressing cell line 3T3-F442A.
Second generation		
Punt CJ et al., 1997 [123]	S9788	Multidrug-resistance-reversing agent S9788 concentrations are achieved in patients at nontoxic doses. Treatment with the combination of doxorubicin and S9788 produced a significant increase in the occurrence of grade 3–4 granulocytopenia.
Saeki T. et al., 2007 [124]	Dofequidar (MS-209)	In patients with advanced or recurrent breast cancer, dofequidar + fluorouracil was well tolerated and is suggested to have efficacy in patients who had not received prior therapy.
Warner et al., 1998 [125]	Dexverapamil	Study of dexverapamil plus anthracycline in patients with metastatic breast cancer who have progressed on the same anthracycline regimen and did not increase anthracycline toxicity.
Nuessler V et al. 1997 [126]	Dexniguldipine	A phase I study using dexniguldipine alone and in combination with vinblastine in patients with metastatic or locally advanced cancer found cardiovascular adverse events such as a blood drop, blood pressure, and decreased heart rate.
Solary E et al., 2000 [127]	Cinchonine	An i.v. infusion of cinchonine might be started 12 h before chemotherapy infusion and requires continuous cardiac monitoring, but no reduction in cytotoxic drug doses.
Kolitz J et al., 2010 [128]	PSC-833 (valspodar)	Randomized phase III trial to compare the effectiveness of combination chemotherapy with or without valspodar followed by interleukin-2 or no further therapy in treating older patients with acute myeloid leukemia. Grade 4 toxicities during IL-2 therapy, included thrombocytopenia and neutropenia, and grade 3 toxicities included anemia, infection, and malaise/fatigue. Low-dose IL-2 maintenance immunotherapy is not a successful strategy in older AML patients. Clinical trial: NCT00006363.
Gandhi L et al., 2007 [129]	VX-710 (biricodar)	Biricodar did not significantly enhance antitumor activity or survival, although minimal toxicity was reported.
Third generation		
Cripe LD et al., 2010 [130]	LY-335979 (zosuquidar)	Erythromycin inhibited in vitro P-gp-mediated transport of both ximelagatran and melagatran and reduced biliary excretion of melagatran in the rats.
Chi K et al., 2005 [131]	OC144-093 (ontogen)	Inhibition of P-gp and multidrug resistance reversal at nM concentrations. No effect on paclitaxel pharmacokinetics. Well tolerated. Toxicities were mainly attributable to paclitaxel (febrile neutropenia).
Kelly RJ et al., 2011 [132]	XR-9576 (tariquidar)	Tariquidar, in combination either with paclitaxel and carboplatin or with vinorelbine, was tested on phase III clinical trials as the first-line therapy in non-small-cell lung cancer patients, but had to be stopped due to the high toxicity observed. Clinical trial: NCT00042302.
Bihorel S et al., 2007 [133]	GF120918 (elacridar)	A phase I and pharmacologic study of elacridar in combination with doxorubicin in patients with advanced solid tumors; elacridar pharmacokinetics were not influenced by coadministration of doxorubicin and produced only minimal side effects at a dose level yielding concentrations able to inhibit the action of P-gp in vitro (hematologic toxicity, namely neutropenia, somnolence, and occasional gastrointestinal complaints). Clinical trial: 2010-020759-30.
Fourth generation		
Yan C et al., 2023 [134]	OY-101	The excellent synergistic anticancer effect with vincristine (VCR) against drug-resistant cells of Eca109/VCR was confirmed using a reversal activity assay.
Yan C et al., 2015 [134]	FD18	Flavonoid dimer FD18 is a new class of dimeric P-gp modulator that can modulate multidrug resistance toward paclitaxel, vinblastine, vincristine, doxorubicin, daunorubicin, and mitoxantrone in human breast cancer LCC6MDR in vitro.
Yu T et al., 2024 [135]	OY-103-B	For the VCR-resistant Eca109 cell line (Eca109/VCR), co-administration of 5.0 μM OY-103-B resulted in a reversal fold of up to 727.2, superior to the typical third-generation P-gp inhibitor tariquidar, and it reversed tumor drug resistance by inhibiting P-gp.
Modulation through signaling pathways		
Wang H., 2016 [60]	Osthole	Reversed P-gp-mediated multidrug resistance by inhibiting the PI3K/Akt signaling cascade.
Hopff S., 2020 [136]	MBR-60	It shows significant apoptotic effects and the mechanism of the compound involves the intrinsic pathway of apoptosis, which demonstrates selectivity for tumor cells over healthy leukocytes.
Li Z., 2024 [137]	ZIF-90@ICG	Impairs mitochondrial functions, downregulating the intracellular ATP level and inhibiting P-gp expression.
Qin K., 2018 [138]	MTX+4-HC	It is mediated through the inhibition of the JAK2/STAT3 signaling pathway.
Wang Y., 2018 [139]	FZD1	FZD1 activation through the Wnt/β-catenin signaling pathway positively regulates the expression of MDR1; therefore, by inhibiting it, it may be possible to reduce the expression and function of P-gp.
Eadie L., 2014 [140]	Imatinib	This TKI is an ABCB1 inhibitor at high micromolar concentrations; however, the clinical relevance of these observations in some studies is limited.
Eadie L., 2014 [140]	Nilotinib	Exhibits a concentration-dependent interaction with ABCB1, acting as a substrate at lower concentrations and as an inhibitor at higher concentrations. The inhibitory effect is observed at concentrations above typical therapeutic levels.
Modulation from a genetic approach		
Wang H., 2016 [60]	Osthole	Inhibited the cellular efflux of P-gp substrates and downregulated the expression of the MDR1 gene.
Tariq I. et al., 2020 [141]	Lipodendriplexes	Demonstrated enhanced cellular uptake, reduced toxicity, and increased gene knockdown efficiency. By silencing MDR1, lipodendriplexes sensitized cancer cells to chemotherapeutic drugs.

Combination chemotherapy presents several theoretical advantages in cancer treatment. By targeting different phases of the cell cycle, it increases the number of cells exposed to cytotoxic effects, thereby reducing the emergence of drug resistance. Furthermore, combination therapy allows for a decrease in the required dose of each individual agent, potentially minimizing side effects and ultimately improving the quality of life for cancer patients [142].

To study the impact of combination therapy on chemoresistance, researchers examined the effects of combining docetaxel and vinblastine on the P-gp-mediated inhibition of apoptosis in non-small-cell lung carcinoma cells. The study demonstrated that co-treatment with both agents significantly reduced the IC50 values for docetaxel and vinblastine, concurrently neutralizing P-gp overexpression. In contrast, treatment with either docetaxel or vinblastine alone resulted in the upregulation of P-gp gene and protein expression. These findings suggest that the observed decrease in P-gp expression levels following combination therapy may represent a crucial mechanism by which this approach overcomes chemoresistance [142].

Building upon these findings, in a study identified nelfinavir and lopinavir, HIV protease inhibitors, as effective sensitizers of highly antimitotic drug-resistant cancer cells. Notably, nelfinavir exerted its sensitizing effect through a mechanism independent of P-gp inhibition. These findings suggest that nelfinavir and lopinavir may possess therapeutic potential as repurposed agents for overcoming drug resistance in cancer treatment [143].

Extending the search for effective strategies to circumvent drug resistance, researchers have explored the potential of inorganic compounds. Cobalt (Co)-salt complexes, exemplified by “salcomine,” have traditionally found applications as oxygen binders and oxidation catalysts. More recently, chiral Co-salt complexes have been used as enantioselective catalysts in asymmetric synthesis. This study focuses on the Co(III)-salt complex MBR-60, synthesized via air oxidation of a Co(II)-salt precursor. In aqueous environments, the axial co-ligands of Co(III)-salt complexes undergo ligand exchange with water molecules resulting in the formation of stable hydroxo complexes. While some Co-salt complexes have exhibited anticancer activity, the authors were intrigued by the lack of investigation into the anticancer potential of the successful tert-butylated and trans-DACH-derived Co-salt complexes. Consequently, they undertook searching into the anticancer activity of MBR-60. Their findings demonstrated that MBR-60 induces apoptosis in Burkitt-like lymphoma and leukemia cell lines, while exhibiting selectivity towards tumor cells over healthy cells. These results underscore the therapeutic potential of cobalt complexes, particularly MBR-60, in the treatment of drug-resistant tumors. Furthermore, the study emphasizes the broader significance of Co-salt complexes as potential therapeutic agents in cancer treatment. MBR-60 exhibits pronounced apoptotic effects in leukemia and lymphoma cells and effectively overcomes daunorubicin resistance in cells overexpressing P-gp. The mechanism of action of the compound involves the intrinsic apoptotic pathway, further demonstrating its selectivity for tumor cells over healthy leukocytes. Overall, these findings strongly suggest that MBR-60 holds significant promise as a potential anticancer agent, particularly in the context of drug-resistant cancers [136].

**Figure 3:**
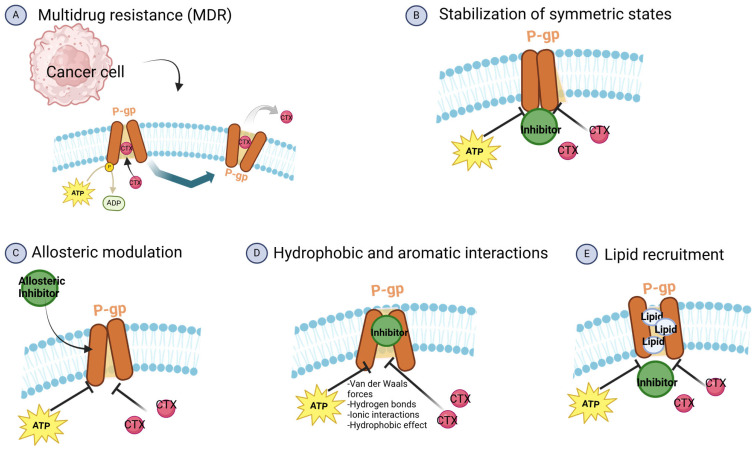
Mechanisms of P-gp Inhibition. (**A**) Multidrug Resistance (MDR): P-gp effluxes a wide range of structurally diverse substrates, including chemotherapy (CTX), leading to decreased drug accumulation and reduced therapeutic efficacy. (**B**) **Symmetric state stabilization:** P-gp inhibitors stabilize the symmetric state of the nucleotide-binding domains, preventing ATP hydrolysis and subsequent drug transport [144]. (**C**) **Allosteric modulation:** Inhibitors bind to P-gp and induce allosteric changes that affect its conformational dynamics and reduce transport function [145]. (**D**) **Hydrophobic and aromatic interactions:** Hydrophobic and π-π (aromatic) interactions between inhibitors and P-gp binding domains are key factors in the inhibitory mechanism. These interactions stabilize the inhibitor-bound conformation of P-gp, hindering its ability to undergo the conformational changes necessary for drug transport [146,147]. Several pharmacophore models have been developed to predict P-gp inhibitory activity. These models often include hydrophobic features and aromatic rings as key components, highlighting the importance of these interactions in the binding process [148,149]. (**E**) **Lipid recruitment:** Certain inhibitors, such as tariquidar, recruit lipid molecules into the P-gp lumen, enhancing inhibitor-induced conformational changes and inhibiting drug transport [150]. The presence of lipids in the membrane may preconfigure inhibitors like tariquidar into active conformations, facilitating their binding to P-gp. This suggests a membrane-assisted mechanism where lipids play a role in the access and binding of inhibitors to P-gp, potentially enhancing their inhibitory effects [151]. Created in BioRender by Morales-Villamil, F. https://BioRender.com/n96f826 (accessed on 21 December 2024).

While originally developed for lung adenocarcinoma, the HA ZIF-90CRZ@ICG nanosystem presents a conceptual framework that could potentially be adapted for overcoming drug resistance in hematological malignancies. This multifunctional nanocomposite, which incorporates hyaluronic acid (HA) for active tumor targeting, zeolitic imidazolate framework-90 (ZIF-90) for drug delivery and inducing mitochondrial dysfunction, crizotinib (CRZ) as a therapeutic agent, and indocyanine green (ICG) for imaging and photodynamic therapy, operates through a mechanism that includes P-gp inhibition. The release of zinc ions (Zn2+) from the ZIF-90 component within the tumor microenvironment leads to mitochondrial dysfunction and ATP depletion, which in turn inhibits P-gp expression, enhancing intracellular drug accumulation. Furthermore, ICG-mediated photodynamic therapy generates reactive oxygen species, contributing to mitochondrial damage and P-gp inhibition. By adapting the targeting component, such as replacing HA with ligands specific to cell surface markers prevalent in drug-resistant blood cancers, this nanosystem’s principles could potentially be translated to improve therapeutic outcomes in hematological malignancies. Further research is necessary to validate this concept and assess its feasibility in blood cancer treatments [137]. This multi-modal approach effectively combines targeted delivery, mitochondrial disruption, P-gp inhibition, and photodynamic therapy, offering a synergistic and potent strategy for combating this challenging clinical problem Figure 3.

In addition to these strategies, natural compounds have emerged as potential candidates for combating drug resistance in acute myeloid leukemia (AML) by inhibiting P-gp activity. Phytol, derived from chlorophyll, has shown promising results in preventing multidrug resistance by modulating P-gp function. Curcumin, a compound found in turmeric, demonstrates efficacy in sensitizing AML and ALL cells to chemotherapy by inhibiting P-gp [152]. Similarly, lupeol, a triterpene present in several fruits and medicinal plants, exhibits P-gp inhibitory effects, thus enhancing drug sensitivity in AML. Heptacosane, a long-chain alkane, also shows promise in combating multidrug resistance by targeting P-gp [64].

Furthermore, the study by Reis et al. (2014) suggests that macrocyclic diterpenes, such as jolkinol D (a macrocyclic diterpene isolated from *Euphorbia piscatoria*) and its derivatives could be valuable in resensitizing multidrug resistance phenotypes by targeting P-gp and other resistance mechanisms simultaneously [153].

This section of the review highlights the ongoing challenges in overcoming P-gp-mediated drug resistance. While direct P-gp inhibition has shown limited clinical success, alternative strategies, including the exploration of natural compounds, the repurposing of existing drugs, and the development of novel nanotechnological approaches, offer promising avenues to overcome drug resistance and improve treatment outcomes in several diseases, including cancer and immune disorders.

### 6.2. Genetic Approach

Genetic approaches to inhibit P-gp primarily focus on manipulating the genes that encode the P-gp protein or its regulatory pathways. One strategy involves gene knockdown or knockout techniques, such as RNA interference (RNAi) or CRISPR/Cas9, to reduce or eliminate the expression of the *ABCB1* gene. By targeting the source of P-gp production, these approaches can potentially enhance the efficacy of chemotherapeutic drugs that are substrates of P-gp by preventing their efflux from cells. Alternatively, modulating regulatory pathways influencing the *ABCB1* expression, such as inhibiting transcription factors like NF-κB or AP-1, which promote P-gp expression, could reduce its levels and activity [104].

Recent advancements in gene editing technology have further empowered researchers to target P-gp directly. CRISPR-Cas9 has emerged as a powerful tool to modify the *ABCB1* gene. Studies have demonstrated that CRISPR/Cas9-mediated knockdown of *ABCB1* can significantly increase the sensitivity of cancer cells to chemotherapeutic agents, such as doxorubicin. For instance, in ovarian cancer cells, CRISPR/Cas9-mediated inhibition of *ABCB1* led to increased drug accumulation and enhanced cell death [154]. Similarly, in osteosarcoma, CRISPR/Cas9-mediated knockout of *ABCB1* restored sensitivity to doxorubicin, highlighting the potential of this approach to overcome drug resistance [155].

Inhibition of P-gp’s ATPase activity by drugs such as zosuquidar, elacridar, and tariquidar is mediated through hydrogen-bond interactions with specific polar residues (Y307, Q725, Y953) within the P-gp drug-binding pocket. Mutating these residues switches inhibition to ATPase activity stimulation. Further analysis revealed that two structural motifs, consisting of a tyrosine residue held by a phenylalanine in a T-shaped aromatic–aromatic interaction, are critical for high-affinity ATP hydrolysis inhibition. Disrupting these motifs by mutation significantly decreased inhibitor-binding affinity. The correlation between the ATPase activity inhibition and the transport function suggests that screening for compounds inhibiting basal ATPase activity could identify high-affinity P-gp modulators and potentially other ABC transporters [156].

Small interfering RNA (siRNA) technology offers a potential solution by silencing the MDR1 gene and reducing P-gp expression. However, effective siRNA delivery to target cells remains a major hurdle. Polyamidoamine dendrimers have been used for efficient siRNA delivery, but their toxicity limits their application. In a study introduced a non-covalent lipid modification to reduce toxicity and optimize the delivery of siRNA targeting *MDR1* using lipid-modified dendrimers, known as lipodendriplexes. These lipodendriplexes demonstrated enhanced cellular uptake, reduced toxicity, and increased gene knockdown efficiency compared to unmodified dendrimers. By silencing *MDR1*, lipodendriplexes sensitized cancer cells to chemotherapeutic drugs, leading to increased drug accumulation and enhanced cell death. Furthermore, the study explored the synergistic effect of combining siRNA-mediated P-gp inhibition with chemotherapy. Sequential treatment with lipodendriplexes followed by a chemotherapeutic agent, such as imatinib mesylate, resulted in significantly increased apoptosis in cancer cells [141].

These findings underscore the pivotal role of the *ABCB1* gene in mediating drug resistance and the promise of CRISPR-Cas9 as a therapeutic strategy to enhance chemotherapy efficacy. These results highlight the limitations of traditional strategies, which have yet to translate into effective clinical outcomes. Precise gene editing, as enabled by CRISPR-Cas9, may offer a transformative benefit in improving chemotherapy efficacy for patients facing multidrug resistance. While these genetic strategies offer potential for increasing the efficacy of chemotherapeutic agents, careful consideration of the potential off-target effects and overall implications for cell function is essential.

### 6.3. Targeting Related Signaling Pathways

Several drugs used in MM, such as doxorubicin, melphalan, dexamethasone, and lenalidomide, have been linked to P-gp-mediated drug resistance. Efforts to reverse this resistance through P-gp inhibition have shown limited success in clinical settings due to pharmacokinetic interactions and lack of specificity of inhibitors. Understanding the biological mechanisms underlying the P-gp overexpression in MM is crucial to identify new therapeutic strategies to combat drug resistance effectively. Further research is needed to explore novel approaches to prevent P-gp overexpression and enhance therapeutic outcomes in MM [47]. It was also demonstrated that cotreatment with vismodegib, a hedgehog pathway antagonist, could sensitize MM cells to carfilzomib, potentially reversing *ABCB1*-mediated drug resistance [50].

The role of P-gp in conferring multidrug resistance is well-established in hematological malignancies. However, the mechanisms underlying P-gp regulation can be complex and vary across different disease contexts. While the primary focus of this review is on hematological malignancies, it is important to acknowledge that insights from other disease areas can inform our understanding of P-gp biology. For example, studies in rheumatoid arthritis and systemic lupus erythematosus have revealed interesting findings regarding the modulation of P-gp expression and function by therapeutic agents. These findings may have implications for the development of novel strategies to overcome drug resistance in hematological malignancies.

Methotrexate (MTX) treatment alone significantly upregulated P-gp expression and mRNA levels in rheumatoid arthritis fibroblast-like synoviocytes (RA-FLSs) compared to control cells. However, the combination of MTX with 4-hydroxycyclophosphamide (4-HC) resulted in lower P-gp expression and mRNA levels. Inhibition of the JAK2/STAT3 signaling pathway using the inhibitor AG490, in conjunction with 4-HC treatment, further suppressed P-gp levels in RA-FLSs. Mechanistic studies revealed that MTX alone increased the production of JAK2 and STAT3 mRNA, as well as elevated levels of phosphorylated STAT3 (p-STAT3) protein. In contrast, the combination of MTX and 4-HC reduced JAK2 and STAT3 mRNA and p-STAT3 protein levels. Pretreatment with AG490 attenuated the MTX- and MTX+4-HC-induced upregulation of JAK2, STAT3, and P-gp. These findings suggest that the enhanced anti-P-gp effect of the MTX+4-HC combination compared to MTX alone in RA-FLSs is mediated through the inhibition of the JAK2/STAT3 signaling pathway. This mechanistic insight may provide a strategy to reverse multidrug resistance in refractory rheumatoid arthritis patients with high P-gp expression. The study contributes to our understanding of P-gp regulation by MTX and supports the potential of combination therapy with low-dose cyclophosphamide to overcome multidrug resistance in rheumatoid arthritis [138].

Additionally, osthole was found to be a potent modulator that could enhance the cytotoxicity of the chemotherapeutic drug doxorubicin in K562/ADM cells, with a reversal efficiency of around 4-fold. Mechanistically, osthole inhibited the cellular efflux of P-gp substrates and downregulated the expression of the *MDR1* gene, which encodes P-gp. Further investigation revealed that osthole reversed P-gp-mediated multidrug resistance by inhibiting the PI3K/Akt signaling cascade. Specifically, osthole decreased the phosphorylation of Akt, which is known to regulate *MDR1* expression [60].

The authors note that this is the first study to examine P-gp activity in systemic lupus erythematosus patients with joint involvement treated with methotrexate. Contrary to their initial hypothesis, the results showed greater P-gp activity in methotrexate responders compared to non-responders. The biological explanation for this finding remains unclear, and the authors suggest that the role of other drug efflux transporters, in addition to P-gp, may influence methotrexate response in systemic lupus erythematosus [94].

Recent research has highlighted the role of the Wnt receptor Frizzled-1 (FZD1) in modulating multidrug resistance in leukemic cells. This study demonstrates that FZD1 overexpression is associated with increased P-gp expression and function, leading to decreased drug sensitivity. Mechanistically, FZD1 activation through the Wnt/β-catenin signaling pathway upregulates MDR1 expression. These findings suggest that targeting FZD1 could be a novel therapeutic strategy to overcome multidrug resistance in leukemia. By inhibiting FZD1, it may be possible to reduce P-gp expression and function, thereby increasing the intracellular accumulation of chemotherapeutic drugs. This could lead to improved treatment outcomes for patients with drug-resistant leukemia [139].

In synoviocytes from patients with rheumatoid arthritis, P-gp activity is decreased with the combination of MTX+4-HC (4-Hydroperoxycyclophosphamide) compared to MTX alone. This effect is mediated by the inhibition of the JAK2/STAT3 signaling pathway [138]. Stimulation of TLR2 leads to the activation of the JAK2/STAT3 signaling pathway. Specifically, the TLR2 agonist Pam3CSK4 increases the expression of PHLDA1 (pleckstrin homology-like domain family, member 1) through a signaling cascade involving JAK2 and STAT3 [157]. It has been proven on several occasions that the JAK/STAT3 pathway can regulate the transcription factor NF-κB in several cell lineages [158,159,160].

Network pharmacology analysis identified NF-κB as a potential target shared by the natural compound solasodine and multidrug resistance in cancer. Solasodine was found to inhibit NF-κB nuclear translocation, leading to the downregulation of P-gp expression in drug-resistant cells. Molecular docking studies confirmed the binding of solasodine to the active site of P-gp, hindering its drug efflux function. Consequently, solasodine increased the intracellular accumulation of chemotherapeutic drugs, such as doxorubicin, in resistant cells. The combination of solasodine and doxorubicin synergistically enhanced cytotoxicity, induced apoptosis, and arrested the cell cycle in P-gp overexpressing multidrug resistant cancer cells. Furthermore, this combination therapy effectively reduced tumor growth and P-gp expression in a xenograft model. These findings highlight solasodine’s potential as a promising natural compound to target NF-κB signaling, downregulate P-gp, and overcome multidrug resistance in cancer, positioning it as a potential fourth-generation P-gp modulator [161].

A publication in 2006 studied the effects of two compounds, oxalyl bis (N-phenyl) hydroxamic acid (OBPHA) and copper N-(2-hydroxy acetophenone) glycinate (CuNG), to overcome drug resistance mediated by P-gp, an efflux transporter that pumps chemotherapeutic drugs out of cancer cells. While this study does not explicitly examine the involvement of canonical signaling pathways such as JAK/STAT or PI3K/Akt in regulating P-gp expression, it provides a valuable example of how cellular processes can indirectly modulate P-gp activity and contribute to drug resistance in leukemia. The study demonstrates that CuNG, by depleting intracellular glutathione, a key antioxidant and cellular redox buffer, significantly impacts P-gp function [162]. Glutathione plays a critical role in maintaining cellular redox homeostasis, and its depletion can lead to oxidative stress. Both contribute to multidrug resistance in cancer cells. P-gp actively pumps chemotherapeutic drugs out of the cell, while glutathione, in conjunction with enzymes such as glutathione S-transferases (GSTs), plays a crucial role in detoxification processes by catalyzing the conjugation of glutathione to a wide variety of xenobiotics, including chemotherapeutic drugs [163,164]. While P-gp and GSTs can be co-expressed in some cancer cells, suggesting potential co-regulation in response to drug resistance, it can also be elevated independently of P-gp [163,165]. For instance, in certain multidrug-resistant cells, GSTs are elevated without a corresponding increase in P-gp, suggesting alternative pathways for drug resistance [164,166]. Interestingly, glutathione has been shown to inhibit P-gp activity in certain experimental settings [167], indicating a potential interaction where glutathione can modulate the activity of P-gp.

## 7. Discussion

P-glycoprotein (P-gp), a pivotal mediator of multidrug resistance in hematological malignancies [47,168,169,170,171,172], functions as an ATP-dependent efflux pump, actively expelling a spectrum of chemotherapeutic agents from malignant hematopoietic cells. This action significantly reduces intracellular drug concentrations, thereby compromising therapeutic efficacy and fostering resistance. Particularly in chronic myeloid leukemia (CML), P-gp overexpression directly correlates with treatment failure [66,70,173], underscoring the necessity for effective modulation strategies in this and other hematological malignancies where P-gp-mediated resistance is a significant obstacle.

The regulation of P-gp expression is a complex interplay of genetic, epigenetic, and signaling mechanisms. Genetic polymorphisms within the *ABCB1* gene, epigenetic modifications such as DNA methylation and histone acetylation, and signaling pathways like JAK/STAT, NF-κB, and Wnt/β-catenin all contribute to the variability of P-gp levels and activity. Notably, NF-κB activation has been demonstrated to directly regulate *MDR1* transcription, highlighting the intricate control of this critical resistance factor. Understanding these regulatory layers is essential for developing strategies to suppress P-gp expression.

Beyond its direct role in drug efflux, P-gp exerts significant immunomodulatory effects within the hematologic microenvironment. Studies have shown P-gp expression in key immune effector cells, including natural killer (NK) cells and CD8+ T cells [6,8], where it influences cytokine secretion and cytotoxic function. In NHL, changes in P-gp expression and function in peripheral blood CD56+ cells have been suggested as potential predictors of chemoresistance. This dual functionality—both in drug resistance and immune modulation—complicates therapeutic targeting. Inhibiting P-gp in anti-tumor immune cells, such as NK cells, could inadvertently suppress beneficial immune responses, necessitating highly targeted approaches.

The tumor microenvironment in hematological malignancies, characterized by complex cellular interactions and cytokine signaling, further modulates P-gp expression and function. Pro-inflammatory cytokines like TNF-α and IL-1β can alter P-gp activity, indirectly affecting drug exposure and creating systemic challenges. Coupled with other factors such as genetic mutations, epigenetic modifications, and alterations in drug metabolism, this necessitates a comprehensive assessment for effective patient management. Recent studies have also highlighted the influence of the gut microbiota on P-gp expression, adding another layer of complexity to therapeutic interventions.

Overcoming P-gp-mediated drug resistance requires a multifaceted approach. Combination therapies, targeting distinct cellular pathways, can synergistically enhance efficacy. Modulating P-gp expression through the inhibition of signaling pathways like JAK/STAT and NF-κB is a promising strategy. Natural compounds such as curcumin, lupeol, and phytol have shown potential in inhibiting P-gp activity, leading to increased intracellular drug accumulation. Developing novel P-gp inhibitors, including second-generation inhibitors like tariquidar and elacridar, and utilizing nanotechnology-based drug delivery systems offer further avenues for improved drug delivery. Genetic approaches, such as siRNA-mediated *ABCB1* gene silencing or CRISPR/Cas9-mediated gene knockout, can effectively reduce P-gp expression, although efficient and safe delivery to target cells remains a major challenge.

While the clinical translation of many direct P-gp inhibition strategies has faced significant limitations due to toxicity, unfavorable pharmacokinetic interactions, and lack of sufficient efficacy in clinical trials [8,9,10,11], it is important to note that substantial progress has nonetheless been achieved in the overall treatment of hematological malignancies over the past decades despite the pervasive challenge of P-gp-mediated resistance. This progress is largely attributable to several factors: the development of novel therapeutic agents, including drugs that are not substrates for P-gp or are potent enough to overcome efflux at therapeutic concentrations; the optimization of multi-agent combination chemotherapy regimens; the successful introduction of targeted therapies (e.g., TKIs, proteasome inhibitors, immunomodulatory drugs) and immunotherapies (e.g., monoclonal antibodies, CAR-T cells), many of which operate through mechanisms distinct from P-gp efflux or are not P-gp substrates; improved diagnostic methods allowing for better risk stratification and personalized treatment approaches; and advancements in supportive care. While P-gp remains a significant barrier for certain drugs and treatment strategies, the development of these alternative and combinatorial strategies has significantly improved remission rates and overall survival in many hematological cancers. Furthermore, while P-gp plays a crucial physiological role in protecting vital organs from xenobiotics and toxins, inhibiting its activity systemically can have unintended consequences, such as altered drug disposition in normal tissues, increased toxicity, and potential immunosuppression. Therefore, balancing therapeutic benefits with potential side effects is essential when considering P-gp modulation. Strategies aimed at selectively targeting P-gp in malignant cells while sparing its function in vital organs and beneficial immune cells are under investigation and represent a critical challenge for future therapeutic design. Addressing P-gp-mediated drug resistance effectively requires a focused and integrated strategy for future research and clinical development.

First, a deeper context-specific understanding of P-gp regulation and function within the diverse microenvironments of hematological malignancies is imperative. This includes further elucidating its complex interplay with signaling pathways, epigenetic modifications, and immune cell subsets, leveraging advanced techniques such as single-cell analysis and spatial transcriptomics to resolve cellular heterogeneity and understand P-gp’s dynamic role within the malignant niche.

Second, the identification and validation of robust predictive biomarkers are crucial for patient stratification. Future efforts should focus on integrating genomic, functional (e.g., P-gp efflux assays in patient samples), and spatial data to identify patients most likely to benefit from P-gp modulation or specific alternative therapies, enabling truly personalized approaches and guiding clinical trial design.

Third, the development of novel therapeutic strategies remains a priority. This encompasses the rational design of next-generation P-gp modulators with improved specificity (e.g., reduced interaction with physiological P-gp, minimal impact on beneficial immune cells) and favorable pharmacokinetic/pharmacodynamic profiles, alongside the exploration of innovative drug delivery systems (e.g., nanoparticles targeting cancer cells) and rational combination therapies designed to bypass or overcome efflux mechanisms based on the specific resistance profile of the tumor. Strategies specifically targeting the interaction between P-gp and immune responses also warrant intensified investigation.

Finally, translating these advancements into clinical practice requires carefully designed trials incorporating biomarker-driven patient selection and evaluating novel P-gp modulation strategies in rational combinations with standard or emerging therapies. Adaptive trial designs and the rigorous collection of clinical outcome data correlated with P-gp status and other biomarkers will be essential to establish the clinical utility of P-gp targeting.

In conclusion, the path forward involves synergistic efforts across basic science, biomarker discovery, therapeutic innovation, and clinical trial design to ultimately neutralize the impact of P-gp on treatment outcomes in hematological malignancies and improve patient survival and quality of life.
